# PYLFIRE: Python implementation of likelihood-free inference by ratio estimation

**DOI:** 10.12688/wellcomeopenres.15583.1

**Published:** 2019-12-10

**Authors:** Jan Kokko, Ulpu Remes, Owen Thomas, Henri Pesonen, Jukka Corander

**Affiliations:** 1 Department of Mathematics and Statistics, University of Helsinki, Helsinki, Finland; 2 Department of Biostatistics, University of Oslo, Oslo, Norway; 3 Parasites and Microbes, Wellcome Trust Sanger Institute, Hinxton, UK

**Keywords:** density-ratio estimation, likelihood-free inference, logistic regression, summary statistics selection

## Abstract

Likelihood-free inference for simulator-based models is an emerging methodological branch of statistics which has attracted considerable attention in applications across diverse fields such as population genetics, astronomy and economics. Recently, the power of statistical classifiers has been harnessed in likelihood-free inference to obtain either point estimates or even posterior distributions of model parameters. Here we introduce PYLFIRE, an open-source Python implementation of the inference method LFIRE (likelihood-free inference by ratio estimation) that uses penalised logistic regression. PYLFIRE is made available as part of the general ELFI inference software http://elfi.ai to benefit both the user and developer communities for likelihood-free inference.

## Introduction

Computer-simulator-based models are becoming increasingly popular across a wide spectrum of scientific applications as they allow a more flexible framework for encoding expert knowledge than typical statistical model families. A simulator model must nevertheless under most circumstances be carefully tuned to produce realistic outcomes when compared with observed data from some process that the simulator is trying to mimic. Likelihood-free inference deals with the need to estimate parameters and quantify uncertainties in parameter values in the light of the observations x when using simulator models. The most common approaches in likelihood-free inference include approximate Bayesian computation (ABC) ^1, 2^ and synthetic likelihood (SL) ^3, 4^. In a general statistical setting, Bayesian inference combines a probability model for the observations x with the parameter(s) *θ* and a prior distribution for the parameters *p*( *θ*) to define the posterior distribution over parameters *θ* as

$$p(\theta |x)=\frac{p(\theta )p(x|\theta )}{p(x)},\;(1)$$

where *p*( *x|θ*) denotes the likelihood function and *p*( *x*) denotes the marginal likelihood defined as *p*( *x*) = ∫ *p*( *θ*) *p*( *x|θ*) *dθ*. The present work considers posterior estimation when the likelihood function is not available, but assuming that synthetic data can be be generated from the model given a configuration of its parameters. Posterior distribution or at least some summaries for it must then be obtained with likelihood-free inference methods, such as ABC or SL. The methods typically use summary statistics *ψ*( *x*) that are engineered to capture the relevant information about the model parameters present in the real or simulated observations *x*.

Recent advances in likelihood-free methods include likelihood-free inference by ratio estimation (LFIRE) ^5^. LFIRE converts the posterior estimation problem into a density-ratio estimation problem that can be solved with logistic regression ^6^. Assume that the dataset $\left\{x_{n}^{\theta }\right\}$ includes *N* observations simulated using particular parameter values *θ* and that $\left\{x_{n}^{m}\right\}$ includes observations simulated from the marginal data distribution, which is obtained by averaging the forward simulation process over random parameter values sampled from the prior distribution *θ ~ p*( *θ*). Logistic regression is then used in LFIRE to model the log-ratio

$$h(x)=\log\frac{p(x\in \{x_{n}^{\theta }\})}{p(x\in \{x_{n}^{m}\})}\;(2)$$

which approximates the log-ratio between the likelihood and marginal likelihood functions evaluated at *θ*. Thomas *et al.* ^5^ propose modelling the log-ratio as a sparse linear combination of summary statistics *ψ _i_* ( *x*) calculated from the observations *x*:

$$h(x)=\beta_{0}+\sum_{i}\beta_{i}\psi_{i}(x)=\beta^{⊤}\psi (x),\;(3)$$

where *β* denotes the linear model parameters and *ψ*( *x*) contains the summary statistics and a constant term. Estimation of the posterior is then based on the idea that we: (1) find the linear model parameters $\hat{\beta }(\theta )$ that minimise the *l*1-penalised logistic loss function evaluated over observation sets $\left\{x_{n}^{\theta }\right\}$ and $\left\{x_{n}^{m}\right\}$, and (2), use the estimated log-ratio model to approximate the posterior density with

$$\hat{p}(\theta |x)=p(\theta )\exp(\hat{\beta }{\left(\theta \right)}^{\text{⊤}}\psi (x)).\;(4)$$

Summary statistics that contribute in the posterior estimation are selected automatically since *l*1 penalisation promotes sparse solutions where the coefficients are forced to zero when the corresponding summary statistic is deemed irrelevant for the prediction: *β _i_* = 0. Since the model for the approximate posterior is estimated based on synthetic observation sets $\left\{x_{n}^{\theta }\right\}$ and $\left\{x_{n}^{m}\right\}$, we can control the dataset size to ensure accurate parameter estimation.

To summarise, LFIRE uses lasso logistic regression to approximate the intractable likelihood function and to achieve data-driven selection of summary statistics in likelihood-free inference. Related works include sparse precision matrix estimation in synthetic likelihood approaches ^7^ and semi-parametric synthetic likelihood ^8^. LFIRE implementations are currently available in MATLAB ^5^ and in ABCpy (Python) ^9^ and the related synthetic likelihood methods in BSL (R) ^10^, while most other likelihood-free inference tools are focussed on ABC and related summary statistic selection methods. General-purpose tools most relevant to the current contribution are reviewed in 11 and available summary statistics selection methods in 12.

The present work introduces a new LFIRE implementation that is compatible with models constructed with the ELFI software ^11^. ELFI is a general-purpose likelihood-free inference software that provides tools for modelling inference problems and includes various ABC approaches. Our PYLFIRE implementation introduced here extends ELFI by adding LFIRE to its pool of available inference methods. PYLFIRE implementation is discussed in closer detail in the next section, after which we provide operational instructions and demonstrate the workflow for estimating a posterior distribution with the software.

## Methods

### Implementation

PYLFIRE generates a marginal or prior predictive observation set $\left\{x_{n}^{m}\right\}$ by sampling *N* configurations from the prior distribution *p*( *θ*) and conditional observation sets $\left\{x_{n}^{\theta }\right\}$ by sampling corresponding observations from the observation model *p*( *x|θ*). The observation sets $\left\{x_{n}^{\theta }\right\}$ are generated with parameter combinations *θ* that indicate the locations in parameter space where lasso logistic regression is used to calculate an approximate log-ratio and approximate posterior based on $\left\{x_{n}^{\theta }\right\}$ and $\left\{x_{n}^{m}\right\}$. Hence the main computational tasks are dataset generation and fitting the logistic regression.

PYLFIRE constructs the simulated datasets with ELFI 0.7.4 ^11^ and estimates the sparse logistic regression model with glmnet 2.1.1 ^13^. ELFI models inference problems as networks that are used in PYLFIRE to generate observations with random parameters that follow the user-specified prior distribution or with fixed parameter values. Logistic regression parameters are estimated with the glmnet implementation that utilises Fortran subroutines for fast execution. Estimation is based on cyclical coordinate descent to minimise the penalised loss function with respect to regression parameters and cross-validation to optimise the penalisation level. When multiple computation cores are available, PYLFIRE can parallellise dataset construction or cross-validation.

### Operation

PYLFIRE requires Python 3.6.0 (or a later version) and a Fortran compiler. However we also provide a Docker container image to run PYLFIRE with the requirements pre-installed. The package and installation instructions are available in ELFI zoo https://github.com/elfi-dev/zoo/tree/master/pylfire. PYLFIRE is provided with a makefile and installation options as follows. To install PYLFIRE in an environment that has Python 3.6 and a Fortran compiler, run installation:


make install


We then recommend testing the PYLFIRE framework with:


make test


The alternative is to build and run the PYLFIRE Docker image:


make docker-build
make docker-run



This requires that Docker is installed, but avoids possible problems with the Fortran compiler. When PYLFIRE is installed, it is available with:


importpylfire


Running posterior inference with LFIRE implemented in the PYLFIRE package then includes (1) ELFI model construction and (2) running LFIRE to estimate posterior probabilities at predetermined parameter combinations. ELFI model construction means that the user adds their parameter priors, simulator, and summarisation rules into a network structure. While model construction does not require observed data, observations can be added in the model. The process is demonstrated in ELFI documentation and tutorials: https://elfi.readthedocs.io/.

ELFI model provides PYLFIRE means to generate observations from the marginal and conditional distributions. In addition to the network model, the user must determine the parameter combinations where approximate posterior is evaluated and the dataset size used in logistic regression. The observed data *x* must also be added in the network model, and the model, parameter combinations, and dataset size are then used to initialise an LFIRE instance that calculates the approximate posterior probabilities. The process is demonstrated in the next section.

### Use case

We demonstrate how ELFI and PYLFIRE can be used to estimate the posterior distribution over model parameters in a lag-one autoregressive model with conditional heteroscedasticity (ARCH(1)). The model describes dependencies between observations in a time series as

$$y^{\left(t\right)}=\theta_{1}y^{\left(t-1\right)}+e^{\left(t\right)}\;(5)$$

$$e^{\left(t\right)}=\xi^{\left(t\right)}\sqrt{0.2+\theta_{2}{\left(e^{\left(t-1\right)}\right)}^{2},}\;(6)$$

where *y* ^(0)^ = 0, and *e* ^(0)^ and *ξ* ^( *t*)^ are independent standard normal random variables. The time series used as observed data in the current example is simulated with parameters ( *θ*_1_, *θ*_2_) = (0.3, 0.7) and has *T* = 100 observations. The simulator is available in the PYLFIRE package:


frompylfire.modelsimportarch


Dependencies between parameters, observations, and summary statistics are described in a predetermined ELFI model that is loaded with:


m = arch.get_model()


**Figure 1.**
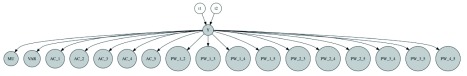
Network structure of the ARCH(1) model.

Here the ARCH(1) simulator parameters are associated with prior distributions *θ*_1_ *~ * ( *–*1, 1) and *θ*_2_ *~ * (0, 1). In our codes we denote the parameters *θ*_1_ and *θ*_2_ as t1 and t2, respectively. The parameters are mapped into time series observations with the simulator, and observations are in turn reduced into summaries that include the time series mean, variance, autocorrelations, and pairwise combinations between the autocorrelations, as described in previous work ^5^. Figure 1 illustrates the network structure of the ARCH(1) ELFI model.

LFIRE determines an approximate posterior distribution over parameter values based on the summaries calculated from an observed time series. At a minimum, the method requires three inputs: an ELFI model with the observed data, candidate parameter combinations, and the dataset size to be used in logistic regression. Parameter combinations must be given as a two-dimensional numpy array where columns correspond to the individual parameters and rows correspond to the parameter combinations to be evaluated. In the current example we estimate posterior probabilities on a 100 × 100 grid over the [ *–*1, 1] *×* [0,1] parameter space:


importnumpyasnp

n  = 100
t1 = np .linspace( -1, 1, n)
t2 = np .linspace( 0, 1, n)
tt1, tt2 = np.meshgrid(t1, t2, indexing ='ij')
params_grid = np .c_[tt1.flatten(), tt2 .flatten()]


and the dataset size used in logistic regression must be given as an integer:


batch_size= 1000


LFIRE is then initialised with:


lfire_method=pylfire.LFIRE(model=m, params_grid=params_grid, batch_size=batch_size)


LFIRE can additionally take precomputed marginal data and custom logistic regression parameters as optional inputs, and allows the user to control whether cross-validation or dataset generation is run in parallel. By default LFIRE generates the marginal data when initialised, uses lasso logistic regression, and runs cross-validation in parallel.

After the LFIRE method is initialised, one can run inference and extract results with:


lfire_res= lfire_method.infer()
lfire_res.summary()

Method: LFIRE
Number of simulations:10000000
MAP estimates: t1:0.434, t2:0.515
Posterior means: t1:0.388, t2:0.654


PYLFIRE also provides two visualisation methods: one for plotting marginal posterior densities and another for plotting pairwise posterior densities:


lfire_res.plot_marginals()
lfire_res.plot_pairs()


**Figure 2.**
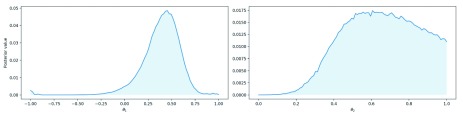
Marginal posterior distributions for parameters *θ*_1_ and *θ*_2_.

**Figure 3.**
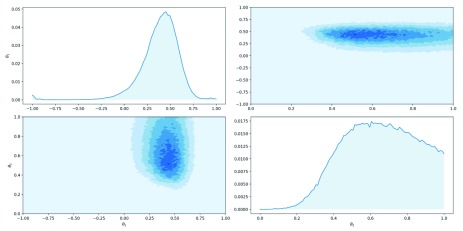
Posterior and marginal distributions for parameters *θ*_1_ and *θ*_2_.


Figure 2 visualises the marginal posterior distributions and Figure 3 visualises the pairwise marginal posterior distributions. PYLFIRE also records the logistic regression parameters estimated at each candidate location so that users can examine how summary statistics were weighted in posterior estimation and determine whether automatic selection focussed on certain summaries. All results are stored in a dictionary and can be extracted with:

lfire_res.results


Finally, LFIRE incorporates automatic summary statistic selection to make posterior estimation robust to irrelevant summary statistics. In this experiment the summary statistics in ARCH(1) model are augmented with 17 white-noise variables:


m_noisy = arch.get_model(noise=17)

lfire_method_noisy = pylfire.LFIRE(model=m_noisy, params_grid=params_grid, batch_size=batch_size)

lfire_res_noisy = lfire_method_noisy.infer()
lfire_res_noisy.summary()

Method: LFIRE
Number of simulations:10000000
MAP estimates: t1:0.475, t2:0.556
Posterior means: t1:0.394, t2:0.64


**Figure 4.**
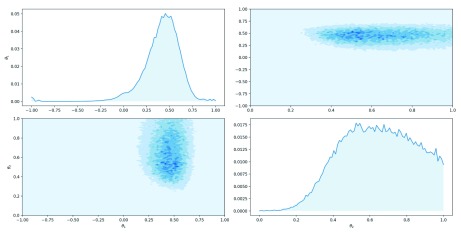
Posterior and marginal distributions for parameters *θ*_1_ and *θ*_2_ when model includes irrelevant summary statistics.

Comparison to previous results confirms that the point estimates and posterior distribution remain about the same despite irrelevant summary statistics. The estimated posterior distribution is visualised in Figure 4.

## Summary

We have introduced PYLFIRE, an open-source Python package running on ELFI and implementing the ratio-estimation-based LFIRE method for likelihood-free inference with automatic summary statistic selection is implemented. PYLFIRE seeks to minimise the computation time in LFIRE with parallelisation and using the external glmnet package ^13^ where key components are written in Fortran. PYLFIRE uses glmnet to fit lasso logistic regression. For convenience, PYLFIRE provides summarised inference results and two built-in plotting methods for visualising the estimated posterior distribution.

## Data availability

### Underlying data

All data underlying the results are available as part of the article and no additional source data are required.

## Software availability

1. Source code available from: https://github.com/elfi-dev/zoo/tree/master/pylfire
2. Archived source code as at time of publication: https://doi.org/10.5281/zenodo.3533332 ^14^.
3. License: BSD 3-Clause
