# Supplementary material for: PYLFIRE: Python implementation of likelihood-free inference by ratio estimation
Source: Wellcome Open Res. 2019 Dec 10;4:197. [Version 1] doi: 10.12688/wellcomeopenres.15583.1 (PMC7041362; doi:10.12688/wellcomeopenres.15583.1)
Supplement: Peer review history

# Peer review: Reviewer response for version 1

**DOI:** 10.21956/wellcomeopenres.17064.r37720

**Published:** 2020-02-24

**Authors:** Guilherme Rodrigues

**Affiliations:** 1 Department of Statistics, University of Brasília, Brasilia, Brazil

**Summary of the paper**

 In modern statistical analysis, the adoption of complex probabilistic models frequently results in likelihood functions that are computationally costly to evaluate ( *intractable*). A vast collection of methods has been engineered to enable statistical inference in such situations. In particular, Approximate Bayesian Computation (ABC) ^1^ and Synthetic likelihood (SL) ^2^ are well established classes of likelihood-free algorithms. As an alternative approach, Thomas *et al.* (2016) ^3^ proposed a novel technique, named LFIRE (likelihood-free inference by ratio estimation), which converts the problem of estimating the posterior distribution as a problem of modeling (by lasso logistic regression fitted over *synthetic/pseudo* samples) the ratio between the data generating distribution and the marginal distribution. A convenient byproduct of such formulation is the resulting semi-automatic selection of summary statistics implied by the lasso (least absolute shrinkage and selection operator) regularization.

 This paper introduces PYLFIRE, an open-source Python implementation of LFIRE that provides for practitioners and developers an easy-to-operate toll for parameter estimation using LFIRE. In an introduction section, the paper describes the general problem of likelihood-free inference in the Bayesian framework and then outline how LFIRE approaches such problem. Next, in a methodological section, it elaborates on the implementation and operational aspects of the package, including details about the installation process and the support offered for parallel computing. A simple bi-dimensional autoregressive model with conditional heteroscedasticity (ARCH(1)) model is then fitted with PYLFIRE for illustrational purposes. To conclude, the paper summarizes the package main features and implementation decisions.

**Major comments:**

 The paper is generally well-written and serves its purpose as a helpful reference source for the PYLFIRE package documentation. The package itself also provides a valuable contribution to the initiative of offering efficient open-source tools that allows immediate exploitation of some of the most advanced solutions for the likelihood-free estimation problem.  

1. Albeit the focus of the paper is, understandably, the computational aspects of the package, it would be beneficial for the reader if the paper was self-contained, in the sense that all information regarding the practical use of the package was present. The description of the LFIRE method, especially, should be meaningfully extended. In this regard, I recommend the inclusion of a pseudo-algorithm that lists the inputs, outputs and the steps involved in the referred approximate posterior sampling algorithm. Moreover, throughout the introduction, a more explicit distinction between *observed* and *synthetic *data should be pursued.
2. It would also be of great use to include some guidance on how to specify the parameters of the algorithm (e.g. the number of simulated samples from the marginal distribution and from the data generating distribution) and to discuss when the method is suitable. For instance, models with a moderate number of parameters or where the prior is diffuse (as compared to the posterior) seem to be out of reach.
3. Considering that another Python routine is currently available, namely, ABCpy, a deeper comparison of the relative merits and deficiencies of each implementation seems necessary. A well-designed numerical simulation would certainly contribute to the robustness of the comparison. 

**Minor comments:**

1. The notation used for the sample sizes is a bit confusing and could be improved. Is there a reason to use lowercase (n) and uppercase (N) letters? In addition, the number of simulated samples from the marginal distribution and from the data generating distribution are necessarily the same?
2. The term “is implemented” should be suppressed from the first paragraph of the Summary section.
3. It is not sufficiently clear how the observed summary statistics are passed on to the function when the user itself provides the simulated data sets.
4. I wonder how feasible it would be to embed the provided functionalities in an optimization process that aims to find the MAP (maximum a posterior probability). If possible, a brief discussion on how to accomplish that could extend the reach of the package when estimating the whole posterior distribution is impracticable.
5. The lasso regression is a reasonable and convenient classifier for estimating the density ratio. However, other classification algorithms could, at least in principle, be employed, either for better accuracy or for computational speed. As such, have the authors considered giving the user control of the classification process, for example, allowing them to pass on a custom python function that returns the estimated classification probabilities (instead of the reported optional “custom logistic regression parameters”)? If feasible, this extension would readily benefit from advances on the probabilistic classification area (e.g. whenever a new package for classification becomes available or in cases where the glmnet 2.1.1 package ^4^ underperforms).

**Conclusions**

 The paper is well-structured, generally clear and represents a valuable addition to the practitioner’s toolbox. For the readers convenience, an extra effort should be made to ensure the paper is self-contained. A list of suggestions was included in the comments above to indicate some options that could be explored in this regard.

I confirm that I have read this submission and believe that I have an appropriate level of expertise to confirm that it is of an acceptable scientific standard.

## References

[1] : Handbook of approximate Bayesian computation. *CRC Press*.2019;

[2] : Bayesian Synthetic Likelihood. *Journal of Computational and Graphical Statistics*.2018;27(1) : 10.1080/10618600.2017.1302882 1-11 10.1080/10618600.2017.1302882

[3] : Likelihood-free inference by ratio estimation. *arXiv*.2016;10.12688/wellcomeopenres.15583.1PMC704136232133422

[4] : Regularization Paths for Generalized Linear Models via Coordinate Descent. *J Stat Softw*.2010;33(1) :1-22 20808728PMC2929880

# Peer review: Reviewer response for version 1

**DOI:** 10.21956/wellcomeopenres.17064.r37335

**Published:** 2020-01-21

**Authors:** Samuel Wiqvist

**Affiliations:** 1 Centre for Mathematical Sciences, Lund University, Lund, Sweden

**Introduction to the topic**

 The problem that is considered in this paper is parameter inference for implicit statistical models (i.e. computer-simulator model) in the Bayesian framework. In particular, what the authors aim at is posterior estimation when the likelihood function is intractable, but data-simulation from the model is available, i.e. what is considered is a likelihood-free inference method. The paper presents the PYLFIRE package ( here), which is an open-source Python package that implements the likelihood-free inference by ratio estimation (LFIRE) method [6]. The LFIRE method is a simulation-based inference method for implicit Bayesian models, where the inference problem is transformed into a density-ration estimation problem, which is solved using Lasso logistic regression [6]. Thus, LFIRE has similarities to approximate Bayesian computation (ABC) [5] and in-particular synthetic likelihood (SL) [4]. However, in contrast to SL, LFIRE does not require any distribution assumptions on the summary statistics [6]. Another feature of the LFIRE method is that it allows for automatic selection of summary statistics by employing Lasso logistic regression [6]. This is an interesting feature since selecting proper summary statistics is one of the main challenges when using ABC in practice. 

**Summary of paper**

 The article starts by introducing the implicit statistical model (i.e., computer-simulator model); in the introduction, the LFIRE method is also briefly introduced. Then the authors explain how PYLFIRE is structured: PYLFIRE extends the ELFI software [1] and utilizes the ELFI framework, and the glmnet 2.1.1 package [2] is utilized for logistic regression modeling. The authors stress that PYLFIRE is developed with performance in mind; therefore, fast FORTRAN subroutines and parallelization are supported. Instructions on how to install PYLFIRE are provided, and a simple case study (simulation study for an ARCH(1) model) highlights the capabilities of PYLFIRE. The paper ends with a summary of the most important features of PYLFIRE.

**Comments**

 The introduction in the paper is quite useful to the reader since it contains a short general introduction to the LFIRE method. However, this could be extended, which would make it easier for a reader not already familiar with the LFIRE method to follow the paper. An idea could be to try to include in the introduction a similar figure as to Figure 1 in [6].

 The authors' reason for developing PYLFIRE could be further processed. Is PYLFIRE developed primarily to extend the capabilities of the ELFI software, to provide an easy-to-use package for LFIRE based inference, or to provide an LFIRE implementation with better computational performance than other implementations?

 Another minor comment is that the authors could include a link to the Jupyter notebook ( here) with the ARCH(1) example in the beginning of the *Use case* section. This would make it easier for a reader to get started using PYLFIRE.

 The authors provide an associated Docker image for PYLFIRE, which is very useful since a user then can circumvent the possibly somewhat involved installation process.

 PYLFIRE implements the LFIRE method [6]; however [6] appears to be still unpublished at the time of writing. It would, therefore, be useful to know if [6] is currently under review in some venue.

 Regarding computational performance: The authors mention that PYLFIRE is implemented with computational performance in mind. Due to this, the authors utilize parallelization and efficient FORTRAN subroutines (via the glmnet 2.1.1 package [2]). However, in the paper, it is not made clear if these efforts of writing efficient code rendered any performance improvements compared to other implementations of the LFIRE method. The authors state that there already exists a MATLAB implementation [6] and another Python implementation (ABCpy [1]) of the LFIRE method. A computational performance comparison with these implementations (or at least the ABCpy implementation) would be of interest, in order to investigate if PYLFIRE indeed has a better computational performance. A comparison with the other implementations could also investigate how easy the packages are to use, and possibly show the reader the potential advantages of using PYLFIRE.

 Furthermore, PYLFIRE utilizes parallelization by default. However, in the paper, the authors do not investigate the potential performance gains from utilizing parallelization. Setting up such a case study is, of course, quite tricky since the results will be highly dependent on the model considered and the particular computer-system used. However, including such a case study could still be of interest since it could highlight: 1) how the user can run the PYLFIRE package in parallel, 2) some idea on what performance gains are plausible. 

 The only case study provided in the paper is a simulation study for the ARCH(1) model. In particular, since computational performance is of interest, would it be interesting to see how the PYLFIRE performs for a more complicated model with a higher-dimensional parameter space. Such a case study could potentially also highlight the parallel computation capabilities of PYLFIRE.

**Conclusion**

 This is an overall clear and well-written paper that for the most parts is easy to follow. However, some parts of the paper could be extended for clarification, and the paper would be improved by including some further analyses (for details, see the *Comments* section). The paper fulfils article approval status - approved, but it could be further improved by incorporating the suggestions presented in the *Comments* section.

I confirm that I have read this submission and believe that I have an appropriate level of expertise to confirm that it is of an acceptable scientific standard.

## References

[1] : ABCpy: A High-Performance Computing Perspective to Approximate Bayesian Computation. *arXiv*.2017;

[2] : Regularization Paths for Generalized Linear Models via Coordinate Descent. *J Stat Softw*.2010;33(1) :1-22 20808728PMC2929880

[3] : Fundamentals and Recent Developments in Approximate Bayesian Computation. *Systematic Biology*.2016; 10.1093/sysbio/syw077 10.1093/sysbio/syw077 PMC583770428175922

[4] : Bayesian Synthetic Likelihood. *Journal of Computational and Graphical Statistics*.2018;27(1) : 10.1080/10618600.2017.1302882 1-11 10.1080/10618600.2017.1302882

[5] : Handbook of approximate Bayesian computation. *CRC Press*.2019;

[6] : Likelihood-free inference by ratio estimation. *arXiv*.2016;10.12688/wellcomeopenres.15583.1PMC704136232133422
